# Interferon‐gamma blocking as a promising treatment for severe liver dysfunction in secondary hemophagocytic lymphohistiocytosis after liver transplantation

**DOI:** 10.1002/jpr3.70126

**Published:** 2025-11-29

**Authors:** Chen Chen, Tao Zhou, Jing Jin, Yi Luo, Lijing Shen, Qiang Xia, Yongbing Qian

**Affiliations:** ^1^ Department of Liver Surgery Reni Hospital Affiliated with Shanghai Jiao Tong University School of Medicine Shanghai China; ^2^ Department of Hematology Renji Hospital Affiliated with Shanghai Jiao Tong University School of Medicine Shanghai China

**Keywords:** emapalumab, Epstein–Barr virus, hematologic disorders, pediatric liver transplantation, sCD‐25

## Abstract

Hemophagocytic lymphohistiocytosis (HLH) is a life‐threatening hyperinflammatory syndrome that can occur after solid organ transplantation but remains underrecognized in this setting. The diagnosis is often delayed due to overlapping clinical manifestations with infection, rejection, or malignancy, and management becomes particularly challenging when accompanied by severe hepatic dysfunction. Here, we describe a pediatric patient who developed Epstein–Barr virus (EBV)–associated secondary HLH following liver re‐transplantation for biliary atresia. On postoperative Day 10, the patient presented with high fever, cytopenia, hypertriglyceridemia, hypofibrinogenemia, and marked hyperferritinemia, accompanied by rapidly worsening liver injury. Bone marrow biopsy confirmed hemophagocytosis. Given the severe hepatic impairment and persistent inflammation, the patient received three doses of emapalumab (1 mg/kg), a monoclonal antibody blocking interferon‐γ, combined with corticosteroids, intravenous immunoglobulin, and antimicrobial prophylaxis. The treatment led to a rapid decline in ferritin levels, reversal of liver dysfunction, and complete clinical recovery without opportunistic infections. The patient has remained stable with normal liver function and negative EBV DNA for over 2 years of follow‐up. This case demonstrates that early interferon‐γ blockade can safely and effectively reverse HLH‐related liver injury after transplantation, offering a promising therapeutic strategy for the management of secondary HLH complicated by severe hepatic dysfunction.

## 1 INTRODUCTION

Hemophagocytic lymphohistiocytosis (HLH) is defined by hyperactivation of immune cells and cytokine overproduction causing multiorgan dysfunction, with diagnosis requiring fulfillment of ≥5HLH‐2004 criteria.^1^ However, HLH is often diagnosed late and remains underrecognized as a hematological complication after transplantation, particularly in patients with organ impairment, a fatal HLH phenotype.^2^ HLH after liver transplantation (LT) remains underestimated, with a mortality rate exceeding 50%.^3^ Herein, we present our experience in treating a pediatric patient who developed Epstein‐Barr virus (EBV)‐associated secondary HLH (sHLH) after re‐LT, with severe liver dysfunction at the time of diagnosis. We aim to raise awareness of HLH as a posttransplant complication and to provide effective therapeutic options.

## 2 CASE REPORT

A child with biliary atresia underwent LT on August 8, 2016, at less than 1 year of age. This patient later developed biliary complications, necessitating re‐LT on July 4, 2023, 7 years after the first LT. The re‐transplant donor was a pediatric patient who had suffered brain death due to traumatic injury. The graft weight was 680 g, with a graft‐to‐recipient weight ratio (GRWR) of 4.25%. Immunosuppression consisted of tacrolimus and corticosteroids.

**Figure 1:**
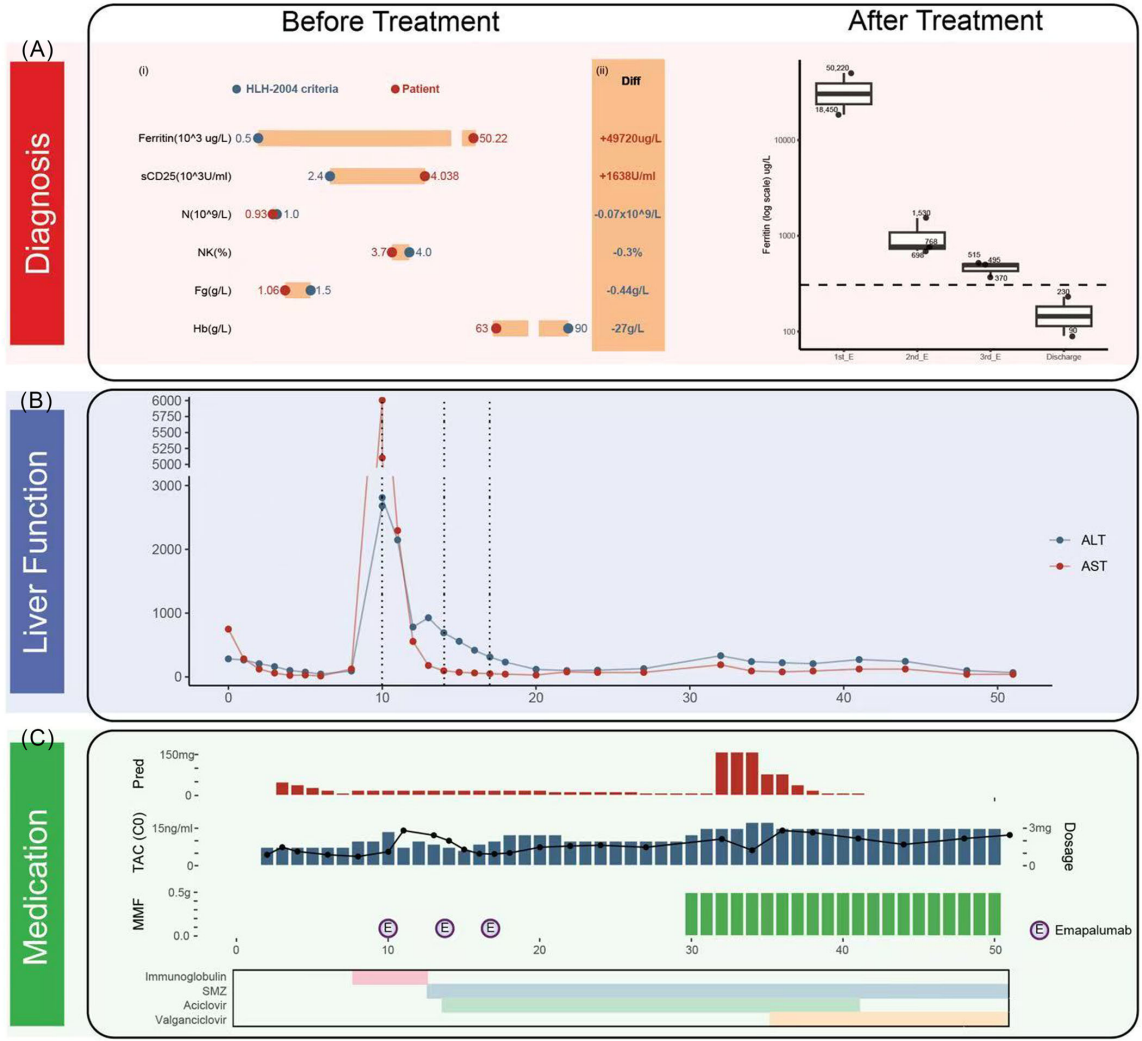
The patient met the HLH‐2004 criteria. Key laboratory findings and treatment course are summarized. (A) Before treatment: lollipop plots show values versus diagnostic thresholds (i); “Diff” indicates how much each exceeded the limit (ii); after treatment: Ferritin declined progressively after three emapalumab doses(dashed line, upper normal limit); (B) liver function changes, with dashed lines marking emapalumab administrations; (C) medication timeline showing prednisone, tacrolimus, and MMF dosage variations. Bars indicate daily tacrolimus dose. Dots show trough concentrations. X‐axis represents postoperative day. Circle “E” mark Emapalumab administrations. The raw data were provided in “Source Data Fig.1.xlsx”. ALT, alanine aminotransferase; AST, aspartate aminotransferase; Diff, difference; E, emapalumab; Fg, Fibrinogen; Hb, Hemoglobin; HLH, hemophagocytic lymphohistiocytosis; MMF, mycophenolate mofetil; N, neutrophil counts; NK, natural killer cells; Pred, prednisone; sCD25, soluble CD25; SMZ, sulfamethoxazole‐trimethoprim; TAC, tacrolimus.

On postoperative Day 6, the patient developed fever, with a peak temperature exceeding 39°C. Infectious etiologies were systematically investigated including respiratory, intra‐abdominal, and bloodstream pathogens by body fluids (peritoneal drain fluid, blood) culture, quantitative PCR, and serology. Investigations revealed mycoplasma pneumoniae IgM (+) and EBV DNA had been continuously positive since 2017 (Table S1). All other common respiratory viruses, *Staphylococcus aureus*, enterobacteriaceae and other bacteria, *Pneumocystis jirovecii*, *Candida albicans* and other fungi were excluded. Peripheral blood tests showed no significant abnormalities in cell morphology. Of note, ferritin levels were markedly elevated. Fever resolved after 3‐day treatment with immunoglobulin and methylprednisolone. However, on postoperative Day 10, the patient's transaminase levels increased sharply (alanine aminotransferase [ALT]: 2680 U/L; aspartate aminotransferase [AST]: 6006 U/L), along with neutrophil reduction (0.93 × 10^9^/L), anemia (hemoglobin, Hb: 63 g/L), hypertriglyceridemia (triglycerides: 4.51 mmol/L; reference: <1.7 mmol/L), hypofibrinogenemia (1.06 g/L; reference: –2.4 g/L) and mildly prolonged coagulation time (PT 13.8 s, reference: 9.4–12.5 s; international normalized ratio 1.23, reference: 0.8–1.15). The ferritin level was extremely high (50,220 μg/L; reference: 11–306.8 µg/L), strongly suggesting sHLH, although further examinations were required (Figure 1).

For symptomatic treatment, the patient received red‐blood‐cell (RBC) transfusion, glutathione, human granulocyte colony‐stimulating factor, and ulinastatin. The dosage of tacrolimus was adjusted and a liver biopsy was performed immediately. Additional tests were arranged: natural killer cells (NK cells) percentage, soluble interleukin‐2 receptor/soluble CD25 (sIL‐2R/sCD25), genetic testing related to familial HLH (fHLH), and bone marrow biopsy.

**Figure 2:**
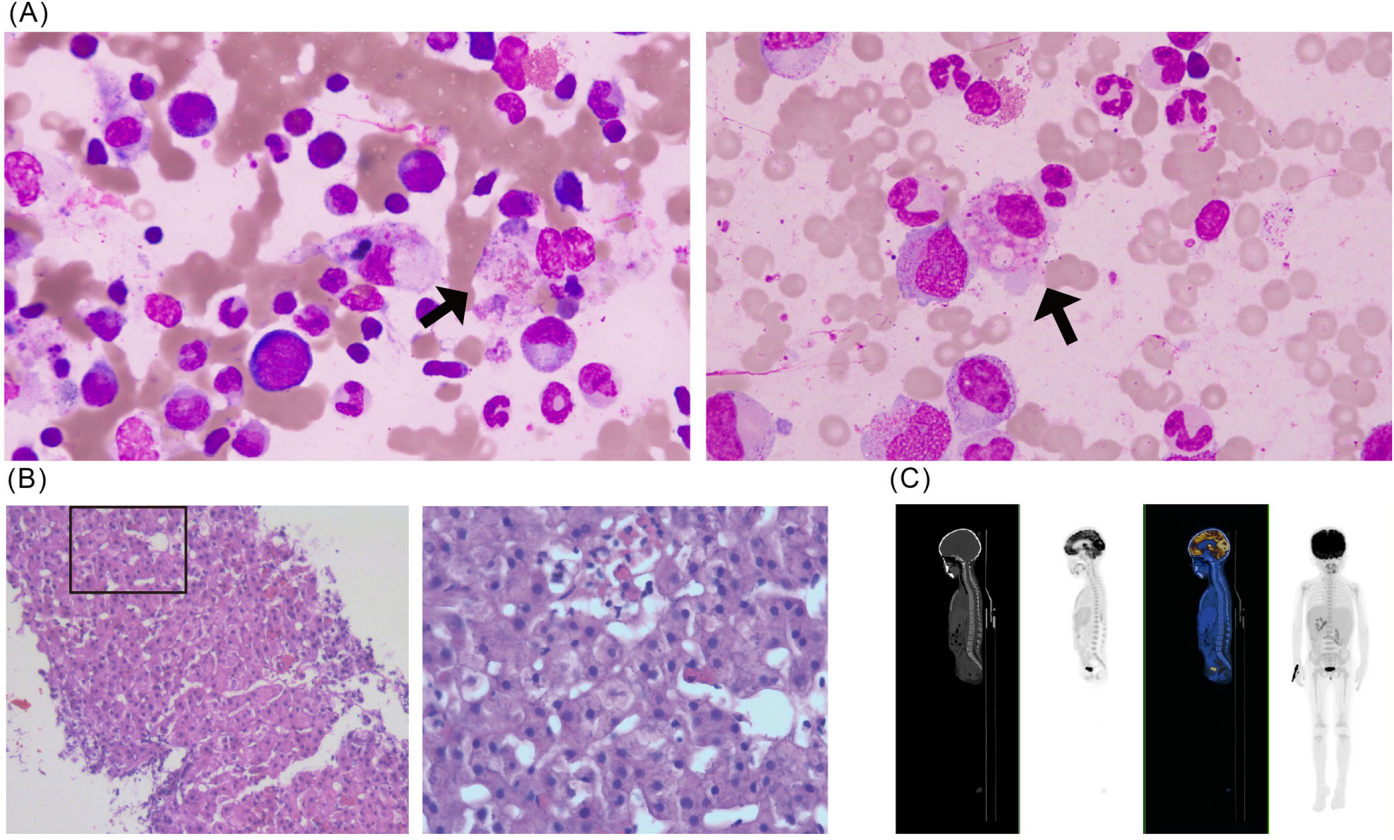
Pathological and radiological evidence supporting the diagnosis of HLH. (A) Bone marrow showed foamy macrophages engulfing blood cells; (B) liver biopsy indicated hepatocytes swelling and ruled out acute allograft rejection; (C) PET‐CT scan showed no evidence of proliferative disorders and lymphoma. HLH, hemophagocytic lymphohistiocytosis; PET‐CT, positron emission tomography‐computed tomography.

After a multidisciplinary consultation with the Department of Liver Surgery, Pediatrics, Hematology, and Infectious Diseases, it was decided to use 20 mg emapalumab (1 mg/kg), a human anti‐interferon (IFN)‐γ monoclonal antibody, to relieve persistent inflammation, along with hepatoprotective therapy.^4^ Given the patient's severe hepatic impairment, we avoided etoposide and instead selected emapalumab, which neutralizes both free and receptor‐bound IFN‐γ and promptly blocks Janus kinase and signal transducer and activator of transcription (JAK‐STAT) signaling. The subsequent examination results confirmed the diagnosis of sHLH. Bone marrow biopsy showed large volume reticular cells with round or oval eccentric nuclei and reticular‐structured chromatin. Most of them have abundant cytoplasm and a foamy appearance without nucleoli. The possibility of EBV‐associated malignancies has also been considered, such as posttransplant lymphoproliferative disorders (PTLDs) and lymphoma, thus, positron emission tomography‐computed tomography scan was performed (Figure 2). Laboratory findings demonstrated decreased NK cell percentage (3.7%) and increased sIL‐2R/sCD25 levels (4038 U/mL). According to the hemophagocytic syndrome diagnostic score (Hscore) (http://saintantoine.aphp.fr/score/),^5^ the patient's probability of having HLH was 99.86%.

During treatment, three doses of emapalumab were administered (postoperative Day 10, 14, and 17), accompanied by immunoglobulin for 5 days, a taper of prednisone, and prophylaxis with acyclovir and valganciclovir. Treatment was effective, with serum ferritin levels rapidly decreasing (Figure 1, Table S2). Mild rejection occurred during the treatment process but was controlled with high‐dose corticosteroids. The patient ultimately recovered completely, with normal bone marrow proliferation, liver function, and ferritin levels. The child was discharged l 50 days after re‐LT, with EBV DNA remaining negative and no recurrence observed during more than 2 years of follow‐up, during which the patient has been monitored every 3 months with biochemical and virological assessments.

## 3 DISCUSSION

This is the first successful case demonstrating the efficacy and safety of emapalumab in salvaging liver injury associated with sHLH. This study aims to provide differential diagnostic approaches for posttransplant HLH and offered treatment strategies for managing complicated sHLH.

This case offers several important lessons. First, suppression the inflammatory storm is crucial for protecting HLH‐induced liver injury. Elevated cytokine levels can exacerbate hepatic inflammation and hepatocytes' death. By blocking upstream IFN‐γ signaling, severe liver damage in this patient was gradually reversed. Thus, controlling inflammation with providing hepatoprotective support is essential for recovery. Emapalumab, a human anti‐IFN‐γ antibody approved for fHLH, has shown survival rate over 70% in a phase II–III trial.^6^ This study fills a gap in understanding its role in sHLH.

Second, no opportunistic infections occurred despite immunosuppressiont, likely due to comprehensive prophylactic measures. Although blocking IFN‐γ may increase infection risk, antimicrobial medications made it manageable. Surprisingly, the chronic active EBV status was cured after treatments.

Third, it is best to initiate treatment as early as possible after the HLH diagnosis. Due to similar manifestations to lymphoma, leukemia, infectious mononucleosis, and severe infections, HLH can easily be misdiagnosed or delayed in diagnosis, sometimes leading to high mortality rates. In this condition, further investigations such as liver biopsies are recommended to rule out allograft rejection. Then timely use of emapalumab can potentially prevent the need for another liver transplant caused by HLH.

More than 70% of HLH patients have varying degrees of liver dysfunction. Survival rates for HLH patients with liver involvement are lower than those without and clinical experience in treating HLH with severe liver damage is limited. Immunosuppression combined with cytotoxic chemotherapy is a traditional method. However, hepatotoxicity caused by chemotherapy requires alternative treatments when prominent liver dysfunction is present. The combination of glucocorticoids, etoposides or fludarabine and gamma globin did not yield satisfactory outcomes in HLH patients with liver failure, with a survival rate of 45.45% in a retrospective study.^7^ Ruxolitinib, a JAK1/2 inhibitor, improved liver injury while reducing macrophages infiltration in the mouse model of HLH.^8^ The IL‐18 binding protein reversed liver damage in a murine model.^9^ Further studies are needed to evaluate the effectiveness of targeted immunomodulation, such as emapalumab, anakinra (a recombinant IL‐1 receptor antagonist), and alemtuzumab (a CD52 monoclonal antibody).^10^ In some cases, liver transplantation or hematopoietic stem cell transplantation may be considered for fHLH with liver injury. To date, current research on liver‐dysfunction‐HLH is limited, and HLH as a hematological complication after LT is still under‐recognized. Among the cytokines implicated in HLH‐associated hyper‐inflammation, IFN‐γ appears to play a central role and has been specifically linked to liver injury.^11^ Therefore, direct neutralization of IFN‐γ represents a mechanistically rational intervention and yielded rapid, durable clinical improvement in our patient.

## 4 CONCLUSION

In conclusion, the use of emapalumab in sHLH patients with severe post‐transplant liver dysfunction in combination with immunoglobin, corticosteroids, immunosuppressants, antimicrobial prophylaxis, and supportive care is effective and safe. Liver injury in HLH patients after transplantation requires special attention.

## ETHICS STATEMENT

This study participant was provided informed consent.

## CONFLICT OF INTEREST STATEMENT

The authors declare no conflicts of interest.

## Supporting information

Source data for Figure 1.

Supplementary Table1. Chronology of EBV DNA in peripheral blood of the patient.

Supplementary Table 2. Cytokine profiles before and after treatment.
