# Supplementary material for: Interferon‐gamma blocking as a promising treatment for severe liver dysfunction in secondary hemophagocytic lymphohistiocytosis after liver transplantation
Source: JPGN Rep. 2025 Nov 29;7(1):14–8. doi: 10.1002/jpr3.70126 (PMC12894085; doi:10.1002/jpr3.70126)
Supplement: Supplementary file 2 — Supplementary Table1. Chronology of EBV DNA in peripheral blood of the patient. [file JPR3-7-14-s001.docx]

**sTable1. Chronology of EBV DNA in peripheral blood of the patient.**

| **Date** | **Months after LT/ Days after re-LT** | **EBV DNA**  **(copies/mL)** |
| --- | --- | --- |
| 2017/5/10 | 9 | 87500 |
| 2017/6/1 | 9 | 33800 |
| 2017/8/16 | 12 | 10800 |
| 2018/1/17 | 17 | 6630 |
| 2018/6/6 | 21 | 15900 |
| 2018/12/6 | 27 | 39600 |
| 2019/4/12 | 32 | 36400 |
| 2019/6/13 | 34 | 8140 |
| 2019/6/19 | 34 | 1100 |
| 2019/7/8 | 35 | 25900 |
| 2019/9/9 | 37 | 12400 |
| 2019/9/24 | 37 | 8860 |
| 2019/11/12 | 39 | 2980 |
| 2019/12/12 | 40 | 9170 |
| 2020/3/13 | 43 | 1490 |
| 2020/6/17 | 46 | 1180 |
| 2020/10/22 | 50 | 912 |
| 2021/3/17 | 55 | 3750 |
| 2021/4/12 | 56 | 6590 |
| 2021/7/19 | 59 | 8110 |
| 2021/10/12 | 62 | 500 |
| 2022/1/25 | 65 | 14000 |
| 2022/10/27 | 74 | 46400 |
| 2022/12/7 | 75 | 3030 |
| 2023/7/14 | 83 months/ 10 days | 1340 |
| 2023/7/19 | 83 months/ 15 days | 1090 |
| 2023/7/28 | 83 months/ 24 days | 1970 |
| 2023/8/28 | 84 months/ 55 days | <400 |
