# Supplementary material for: Interferon‐gamma blocking as a promising treatment for severe liver dysfunction in secondary hemophagocytic lymphohistiocytosis after liver transplantation
Source: JPGN Rep. 2025 Nov 29;7(1):14–8. doi: 10.1002/jpr3.70126 (PMC12894085; doi:10.1002/jpr3.70126)
Supplement: Supplementary file 3 — Supplementary Table 2. Cytokine profiles before and after treatment. [file JPR3-7-14-s003.docx]

**sTable 2. Cytokine profiles before and after treatment.**

|  | IL-1β  (pg/mL) | IL-2R  (U/mL) | IL-6  (pg/mL) | IL-8  (pg/mL) | IL-10  (pg/mL) | TNFα  (pg/mL) |
| --- | --- | --- | --- | --- | --- | --- |
| Before treatment | ＜5 | 3171 | 9.63 | 10.9 | 164 | 24.4 |
| After 1st Emapalumab | 31.4 | 2307 | 2.1 | 49.9 | 32.3 | 41.8 |
| After 2nd Emapalumab | 9.13 | 1610 | 2.6 | 34.8 | 28.4 | 22.4 |
| After 3rd Emapalumab | ＜5 | 799 | 2.74 | 31.9 | 11.6 | / |
